# Supplementary figures and images for: Annexin A1 as a key modulator of lung inflammation during coronavirus infections
Source: Clin Sci (Lond). 2025 Nov 17;139(22):1489–505. doi: 10.1042/CS20255801 (PMC12751058; doi:10.1042/CS20255801)

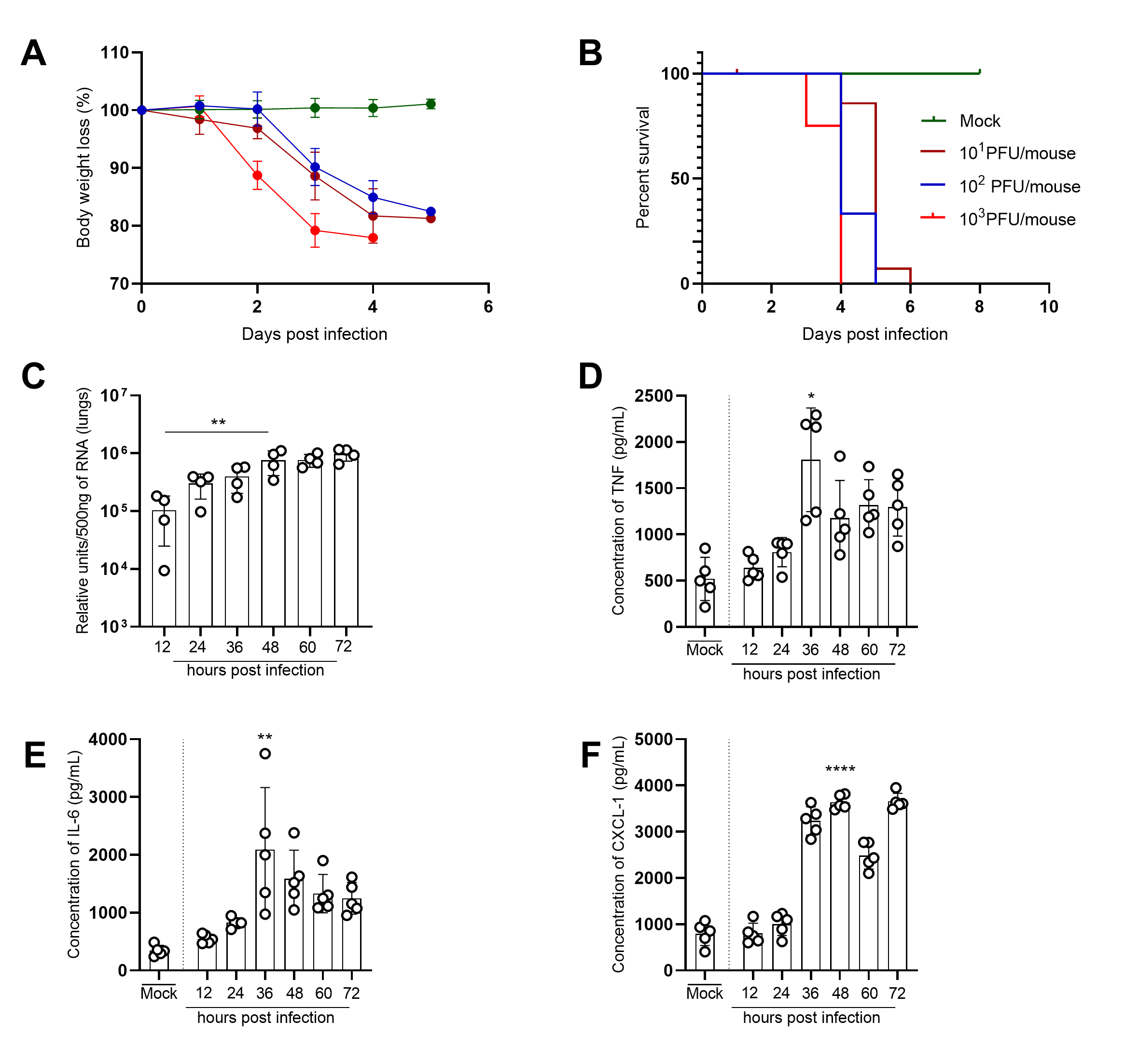

Supplement: Online supplementary figure 1 [file CS-139-22-CS20255801-s001.tif]
